# Chronic HIV-1 Infection Frequently Fails to Protect against Superinfection

**DOI:** 10.1371/journal.ppat.0030177

**Published:** 2007-11-16

**Authors:** Anne Piantadosi, Bhavna Chohan, Vrasha Chohan, R. Scott McClelland, Julie Overbaugh

**Affiliations:** 1 Division of Human Biology, Fred Hutchinson Cancer Research Center, Seattle, Washington, United States of America; 2 Department of Pathobiology, University of Washington, Seattle, Washington, United States of America; 3 Department of Medical Microbiology, University of Nairobi, Nairobi, Kenya; 4 Department of Medicine, University of Washington, Seattle, Washington, United States of America; 5 Department of Epidemiology, University of Washington, Seattle, Washington, United States of America; National Institutes of Health, United States of America

## Abstract

Reports of HIV-1 superinfection (re-infection) have demonstrated that the immune response generated against one strain of HIV-1 does not always protect against other strains. However, studies to determine the incidence of HIV-1 superinfection have yielded conflicting results. Furthermore, few studies have attempted to identify superinfection cases occurring more than a year after initial infection, a time when HIV-1-specific immune responses would be most likely to have developed. We screened a cohort of high-risk Kenyan women for HIV-1 superinfection by comparing partial *gag* and *envelope* sequences over a 5-y period beginning at primary infection. Among 36 individuals, we detected seven cases of superinfection, including cases in which both viruses belonged to the same HIV-1 subtype, subtype A. In five of these cases, the superinfecting strain was detected in only one of the two genome regions examined, suggesting that recombination frequently occurs following HIV-1 superinfection. In addition, we found that superinfection occurred throughout the course of the first infection: during acute infection in two cases, between 1–2 y after infection in three cases, and as late as 5 y after infection in two cases. Our results indicate that superinfection commonly occurs after the immune response against the initial infection has had time to develop and mature. Implications from HIV-1 superinfection cases, in which natural re-exposure leads to re-infection, will need to be considered in developing strategies for eliciting protective immunity to HIV-1.

## Author Summary

Superinfection with HIV-1 occurs when an individual infected with one strain of HIV-1 acquires a second strain, from a different partner. There are more than 20 published cases of HIV-1 superinfection. These cases have raised concerns for HIV-1 vaccine design because they indicate that the immune response generated against natural infection is not always sufficient to protect against later exposures to the virus. However, it remains unclear how often HIV-1 superinfection occurs, especially at times in infection after an immune response would be expected. We investigated the incidence of HIV-1 superinfection in a cohort of 36 high-risk women followed for approximately five years after their first HIV-1 infections. We found seven cases of HIV-1 superinfection. Five cases occurred more than a year after the initial infection, a time when the immune response would have had time to develop and broaden. In three cases, the initial and superinfecting viruses were classified as the same HIV-1 genetic subtype, indicating a lack of protection against closely related viruses. Our results suggest that natural HIV-1 infection does not always elicit a protective immune response, an important consideration in developing strategies for HIV-1 vaccine design and testing.

## Introduction

HIV-1 superinfection (also referred to in the literature as re-infection) has been described in more than 20 cases [1–5], demonstrating that natural infection with HIV-1 does not always generate a protective immune response. Evidence of superinfection presents a potential challenge to HIV-1 vaccine design because most of the other viruses for which vaccines exist do elicit protective immunity in natural infection. However, the implications of HIV-1 superinfection for vaccine design remain controversial, in part because it is unclear how often superinfection occurs and whether it is restricted to times in infection when an HIV-specific immune response has not yet developed, such as soon after the first infection, or has become impaired, such as during advanced AIDS.

To date, several population-based studies have measured the incidence of HIV-1 superinfection, with conflicting results. At one extreme, two studies found that superinfection occurred at a rate close to that of initial infection: 4% per year among women in Kenya [1], compared to an initial infection rate of 8% per year in the same cohort [6]; and 5% per year among men in southern California [2], compared to an initial infection rate of 5% per year in a comparable cohort [7]. At the other extreme, two studies did not identify any cases of HIV-1 superinfection despite extensive follow-up [8,9]. Differences in study design and methodology may account for some variability in the detection of superinfection. It is also likely that the incidence of HIV-1 superinfection depends on the frequency of re-exposure and potentially on characteristics of the viruses being studied.

One factor that may influence the incidence of HIV-1 superinfection is the relatedness of the virus strains. In approximately half of the published cases of superinfection, the initial and superinfecting viruses belonged to different subtypes, which can differ by ∼30% in the *envelope* gene. In contrast to these cases of intersubtype superinfection, in cases of intrasubtype superinfection viruses differ by ∼10% or less in *env* [10] and might be expected to share antigenic properties. Intrasubtype superinfection is more difficult to detect and has primarily been documented for subtype B viruses [2,11–16]. It is not known how often intrasubtype superinfection occurs in regions where non-subtype B viruses predominate, and these are the regions where HIV-1 is most prevalent.

The rate of HIV-1 superinfection may also depend on whether an HIV-specific immune response has been generated at the time of exposure to the second virus. It is possible that superinfection occurs more frequently in the period soon after the initial infection, before the development of an HIV-1-specific immune response, although there are reports that indicate this is not always the case [12,17,18]. The cytotoxic T lymphocyte (CTL) response generally arises within the first month after infection [19], presenting the first potential barrier to HIV-1 superinfection. Experimental superinfection of macaques with HIV-2 demonstrated that superinfection was limited to the first month after infection in that model [20]. However, HIV-1 superinfection in humans has been documented to occur after the development of a CTL response [12,16,18,21], including one case in which CTLs recognized the superinfecting strain [12].

The other arm of the adaptive immune response to HIV-1, neutralizing antibodies, does not arise until later in infection but may play a stronger role in preventing superinfection. Neutralizing antibodies have been shown to block primary infection in an experimental animal setting [22–25]. Preliminary evidence suggests that they may also block superinfection; Smith et al. recently found that three individuals who became superinfected had weaker neutralizing antibody responses to their initial infections than did control individuals [26]. However, neutralizing antibodies generally do not arise until 2 mo after infection, and antibodies do not broaden to recognize heterologous strains until approximately 1 y after infection, if at all [27]. Because most of the documented cases of superinfection occurred within the first year after infection [11], it is tempting to speculate that antibodies could protect against later superinfections. However, in many studies, individuals were not followed for longer than a year. Studies with long follow-up are needed to determine whether later superinfections occur despite the opportunity for the host to develop a broad immune response.

Finally, most of the previous studies are likely to have underestimated the incidence of superinfection because they examined only one region of the HIV-1 genome [1,2,9] or two neighboring regions [8]. This approach may have missed cases of superinfection in which the initial and superinfecting viruses recombined, a frequent event during HIV-1 replication [28]. Examining more than one region of the HIV-1 genome may provide a better estimate of the incidence of HIV-1 superinfection.

To address these issues, we screened a prospective cohort of Kenyan women for HIV-1 superinfection. We compared sequences from two non-neighboring regions of the genome (*gag* p17 and *env* V1-V5) at time points soon after the initial infection and approximately five years later. We identified seven cases of HIV-1 superinfection among 36 women. Three of these cases were subtype A intrasubtype superinfections. In the other four cases, the initial and superinfecting viruses belonged to different subtypes in at least one of the genome regions examined. In five out of seven cases, the superinfecting strain was only detected in one region of the genome (*gag* or *env*), indicating that recombination frequently occurred following superinfection. We also defined the timing of each superinfection case, and these results indicate that superinfection can occur as late as five years after the initial infection.

## Results

To identify potential HIV-1 superinfection cases, we analyzed HIV-1 proviral sequences from 36 individuals at two time points. The first (“initial”) time point was a median of 111 days post-infection (DPI) (range 17–338), and the second (“chronic”) was a median of 1,901 DPI (range 1,309–2,631, which corresponds to 3.6–7.2 y post-infection). For each individual, we studied a median of seven *gag* sequences (range 3–19) and eight *env* sequences (range 3–13) from the initial time point and a median of seven *gag* sequences (range 5–13) and seven *env* sequences (range 4–12) from the chronic time point.

**Figure 1:**
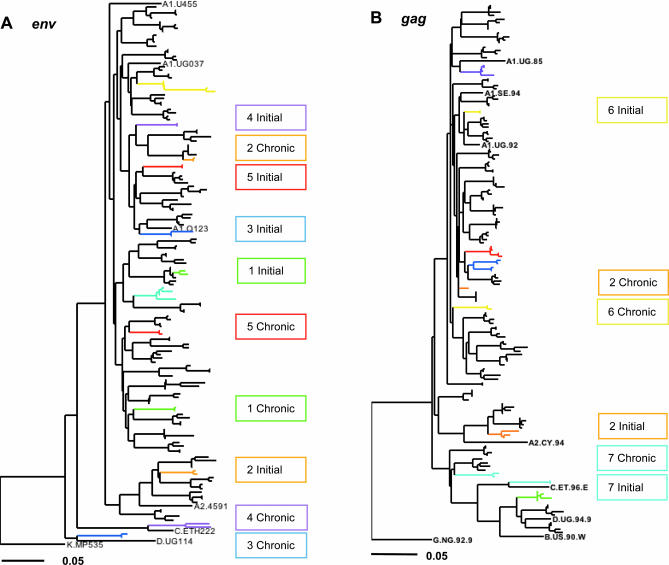
Maximum-Likelihood Phylogenetic Trees of *env* (A) and *gag* (B) Sequences from 36 Individuals Each tree contains two initial and two chronic sequences from each individual (unlabeled branches) that were selected to represent both strains in presumed superinfection cases and were selected randomly for other cases. Reference sequences representing different subtypes from the LANL database are also included (labeled branches). Cases that demonstrate separate clustering on either or both trees are highlighted in color. In most cases, separate clustering is evident in only *env* or only *gag*. Sequences from each of these presumed superinfection cases are labeled with the case number and either “initial” or “chronic” on the tree(s) that demonstrates separate clustering. Green = Case 1, orange = Case 2, blue = Case 3, purple = Case 4, red = Case 5, yellow = Case 6, light blue = Case 7. Bootstrap values were omitted for clarity; however, except for the superinfection cases, sequences from each individual form a monophyletic cluster with ≥82% bootstrap support (most have 100% bootstrap support).

In examining sequences from the initial time point, sequences from each individual formed a monophyletic cluster in both *gag* and *env* phylogenetic trees (unpublished data), suggesting that all individuals were initially infected from a single source partner (i.e., individuals were not coinfected). The initial *env* sequences had a median pairwise diversity of 2.6% (range 0%–6.4%) and a median divergence from their most recent common ancestor (MRCA) of 1.6% (range 0%–11.5%). The initial median *gag* diversity and divergence were 0.3% (0%–1.3%) and 0.6% (0%–3.3%), respectively. In nine individuals, the initial *gag* sequences and *env* sequences belonged to different HIV-1 subtypes, indicating initial infection with intersubtype recombinant viruses. Figure 1 shows *env* (A) and *gag* (B) maximum-likelihood phylogenetic trees containing four representative sequences (two initial and two chronic) from each of the 36 individuals. All initial *env* V1–V5 sequences clustered with subtype A reference sequences (including both sub-subtypes, A1 and A2), which was a selection criterion for this study. However, initial *gag* p17 sequences clustered with subtype C in one individual and subtype D in three individuals and were A1/D recombinant in two individuals and A2/D recombinant in three individuals.

Through analysis of sequences from both the initial and chronic time points, we identified seven potential cases of superinfection, in which some or all of the chronic sequences clustered separately from the initial sequences on phylogenetic trees of *env*, *gag*, or both. These cases are highlighted in color on the phylogenetic trees in Figure 1. Separate clustering was observed in both *env* and *gag* trees for one case (Case 2), in only *env* for four cases (Cases 1, 3, 4, and 5), and in only *gag* for two cases (Cases 6 and 7).

In three of these cases, the chronic sequences belonged to a different subtype than the initial sequences, indicating intersubtype superinfection. Specifically, as shown in Figure 1A, the initial *env* sequences from Case 3 are subtype A while the chronic sequences are subtype D. The initial *env* sequences from Case 4 are subtype A, while the chronic sequences are subtype C. As shown in Figure 1B, the initial *gag* sequences from Case 7 are subtype C, while the chronic sequences cluster between subtypes A and C and are an A/C recombinant (Figure S1). One of the remaining cases was an inter-sub-subtype superinfection: the initial sequences from Case 2 were sub-subtype A2 in *env* and D/A2 recombinant in *gag* (Figure S2A), while the chronic sequences were sub-subtype A1 in *env* and D/A1 in *gag* (Figure S2B). In the remaining three cases (Cases 1, 5, and 6), both the initial and chronic sequences clustered with subtype A reference sequences, indicating intrasubtype superinfection.

**Figure 2:**
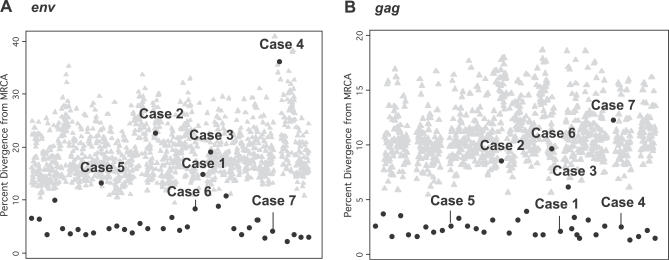
Maximum Divergence from the MRCA for Chronic *env* (A) and *gag* (B) Sequences Comparisons within individuals (black circles) and between subtype A–infected individuals (gray triangles) are arbitrarily distributed along the *x*-axis. Superinfection cases are labeled in both plots. For most cases, the high divergence indicative of superinfection is apparent in one region of the genome but not the other, consistent with Figure 1.

Quantitative analysis of virus divergence supported superinfection in these cases. For each individual, we calculated divergence as the maximum genetic distance between any chronic sequence and the MRCA of the initial sequences. To determine what level of divergence would be expected in an intrasubtype superinfection, we also measured the divergence between each pair of individuals who had a subtype A virus (including sub-subtypes A1 and A2). Figure 2 shows plots of *env* (A) and *gag* (B) divergence within and between individuals; the potential superinfection cases from Figure 1 are labeled. Excluding these presumed superinfection cases, the divergences within each individual ranged from 2.1% to 10.7% in *env* (median = 4.5%) and from 1.2% to 3.9% in *gag* (median = 2.3%). The between-individual divergence ranged from 10.4% to 38.2% (median = 17.9%) in *env* and from 5.4% to 17.1% (median = 10.1%) in *gag*. These ranges are somewhat higher than what is typically reported [10,29,30] because we included comparisons between sub-subtype A2 and sub-subtype A1 sequences. Furthermore, we calculated maximum divergence rather than average diversity in order to identify potential superinfection cases in which just one sequence belonged to the superinfecting strain. As shown in Figure 2, the levels of divergence observed in the three potential intrasubtype superinfection cases (Cases 1, 5, and 6) are in the range that would be expected for a superinfection. Consistent with the results of phylogenetic analysis (Figure 1), Cases 1 and 5 demonstrate high divergence in *env* but not *gag*, while Case 6 demonstrates high divergence in *gag* but not *env*. Case 2, the inter-sub-subtype superinfection that was detected in both *env* and *gag* shows high divergence on both plots. The divergence in the intersubtype superinfection cases (Cases 3 and 4 in *env* and Case 7 in *gag*) is higher than in the intrasubtype cases, as would be predicted.

For all seven potential cases of HIV-1 superinfection identified by phylogenetic analysis, we examined samples from intervening time points to estimate when superinfection occurred. As described below, we examined samples using both single-copy sequencing and strain-specific PCR (ssPCR), which allowed us to sample a greater number of genome copies. For cases of superinfection in which the second strain was initially detected in only *env* or *gag*, this approach also allowed us to carefully screen the other region at intervening time points. In one case (Case 1), we detected a second strain in *gag* at an intervening time point, whereas it was not found in our initial screen. The timing and characterization of superinfection cases are summarized in Table 1.

**Table 1:**
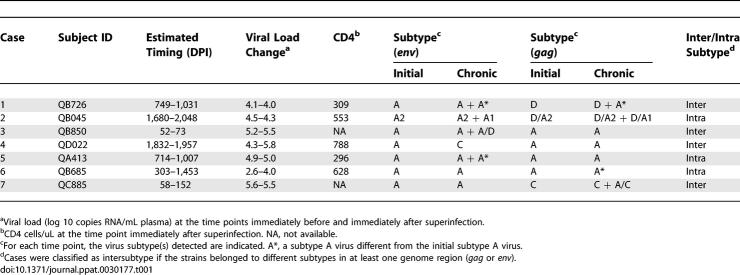
Summary and Estimated Timing of HIV-1 Superinfection Cases

### Case 1 (QB726): Intrasubtype Superinfection (Subtype A) between 749 and 1,031 DPI Detected in *env* and *gag*

**Figure 3:**
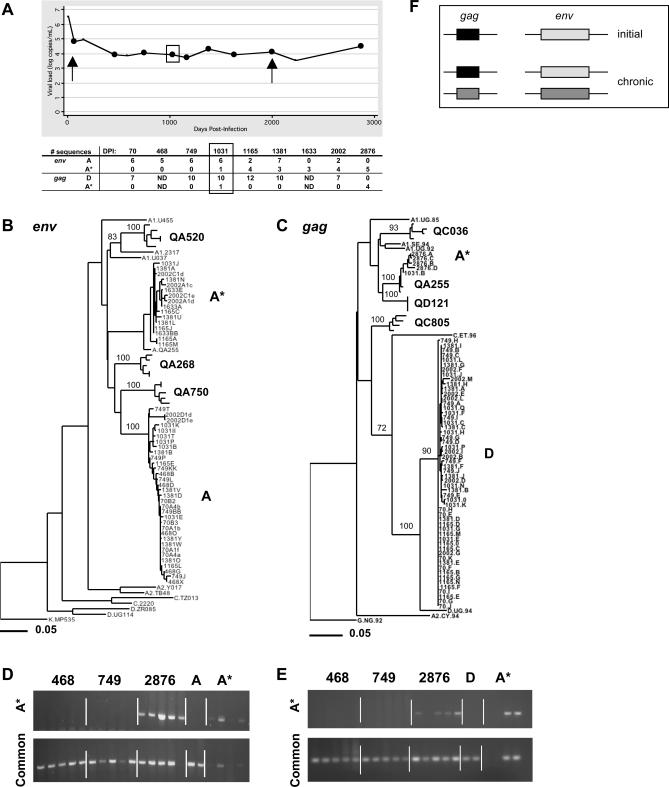
Case 1 (QB726): Intrasubtype Superinfection (Subtype A) between 749 and 1,031 DPI Detected in *env* and *gag* (A) Plot of viral load (log RNA copies/mL plasma) versus DPI. Sequences were obtained by single-copy PCR from time points marked by circles. Arrows indicate the initial and chronic time points used in the analysis in Figure 1, and the box indicates the first time point at which the superinfecting strain was detected. The number of sequences of each strain at each time point is indicated in a table below the graph. ND, not done. (B) Maximum-likelihood tree of *env* sequences from all time points. Sequence names from QB726 are comprised of the sample DPI and a unique identifier. The cluster of strain A contains sequences from all time points except 1,633 and 2,876 DPI. The cluster of strain A* contains sequences from 1,031, 1,165, 1,381, and 2,002 DPI. Sequences from three other individuals (QA520, QA268, and QA750), as well as subtype reference sequences from the LANL database, are also included. Bootstrap values greater than 70% are shown. (C) Maximum-likelihood tree of *gag* sequences from all time points. Sequence names from QB726 are comprised of the sample DPI and a unique identifier. The cluster of strain D contains sequences from all time points except 2,876 DPI. The cluster of strain A* contains sequences from 1,031 and 2,876 DPI. Sequences from four other individuals (QC036, QA255, QD121, and QC805), as well as subtype reference sequences from the LANL database, are also included. Bootstrap values greater than 70% are shown. (D) Amplification of 50 HIV-1 genome copies from the indicated time points using *env* strain–specific primers (top panel) or *env* common primers (bottom panel). For each time point, each lane contains an estimated ten HIV-1 genome copies of template input. As a control, we also amplified 10^3^ copies of a cloned A sequence (two lanes) and one copy of a cloned A* sequence (four lanes). (E) Amplification of 50 HIV-1 genome copies from the indicated time points using *gag* strain–specific primers (top panel) or *gag* common primers (bottom panel). For each time point, each lane contains an estimated ten HIV-1 genome copies of template input. We also amplified 10^3^ copies of a cloned D sequence (two lanes) and one copy of a cloned A* sequence (four lanes). (F) Summary of the strains detected in each genome region at the initial time point and during chronic infection (all other time points). Light gray = subtype A; dark gray = A*; black = subtype D.

For Case 1, intrasubtype superinfection was first suggested by separate clustering of initial and chronic *envelope* sequences on a phylogenetic tree (Figure 1). *Env* sequences from 70 DPI formed a monophyletic cluster (strain A), while some sequences from 2,002 DPI clustered separately (strain A*). To determine when strain A* first appeared, we examined multiple sequences from six intervening time points. These time points are highlighted in Figure 3A, a plot of this individual's viral load throughout infection. A phylogenetic tree of all *env* sequences is shown in Figure 3B. *Env* sequences from 468 DPI and 749 DPI clustered with strain A sequences. Among the sequences from 1,031 DPI, one clustered with strain A*, suggesting that superinfection occurred between 749 and 1,031 DPI. We detected both strain A and strain A* at 1,165, 1,381, and 2,002 DPI, and we detected only strain A* in sequences from a later sample (2,876 DPI). The number of sequences of each strain at each time point is listed in Figure 3A.

We used ssPCR of *env* sequences to assess the presence or absence of strain A* at time points before it was detected by single-copy sequencing. This method allowed us to sample a greater number of genome copies (50 copies per time point versus ∼6). Using primers specific for strain A*, we did not detect the new strain in 50 genome copies from 468 or 749 DPI but did detect it at 2,876 DPI, consistent with results from the sequence analysis (Figure 3). The 70-DPI sample was not included in this analysis because we did not have enough DNA available.

In our initial analysis of *gag* sequences from Case 1, we did not detect a superinfecting strain at 2,002 DPI. As shown in Figure 1, sequences from 70 and 2,002 DPI formed a monophyletic cluster within subtype D. To determine whether a superinfecting strain could be detected in the *gag* region at intervening time points, we examined a total of 47 single-copy sequences from five samples (listed in Figure 3A). At 1,031 DPI, we found one sequence that clustered with subtype A sequences (A*) (Figure 3C). The subtype A strain was not detected by single-copy sequencing at 1,165 or 1,381 DPI but was the only strain present at 2,876 DPI. Using strain-specific *gag* primers, we did not detect the subtype A strain in 50 genome copies from 468 or 749 DPI but did detect it at 2,876 DPI (Figure 3E).

As summarized in Figure 3F and Table 1, these results suggest that this individual was initially infected with an intersubtype recombinant strain, containing subtype A in *env* and subtype D in *gag*. Between 749 and 1,031 DPI, this individual acquired a second strain that belonged to subtype A, which was detected in both *env* and *gag* sequences, although less consistently in *gag*.

### Case 2 (QB045): Intrasubtype Superinfection (Sub-Subtype A2–A1) between 1,680 and 2,048 DPI Detected in *env* and *gag*

**Figure 4:**
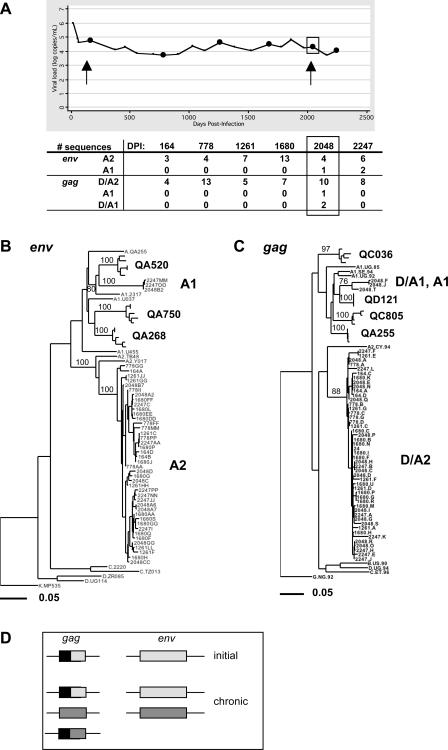
Case 2 (QB045): Intrasubtype Superinfection (Sub-Subtype A2–A1) between 1,680 and 2,048 DPI Detected in *env* and *gag* The layout of this figure is similar to that of Figure 3, as described in the legend for Figure 3. (A) Plot of viral load (log RNA copies/mL plasma) versus DPI with the number of sequences of each strain indicated. (B) Maximum-likelihood tree of *env* sequences from all time points. The A2 cluster contains sequences from all time points, while the A1 cluster contains sequences from 2,048 and 2,247 DPI. (C) Maximum-likelihood tree of *gag* sequences from all time points. The cluster of strain D/A2 contains sequences from all time points. The cluster of strains D/A1 and A1 contains sequences from 2,048 DPI. (D) Summary of the strains detected. Light gray = sub-subtype A2; dark gray = sub-subtype A1; black = subtype D.

For Case 2, intrasubtype superinfection was suggested by separate clustering of initial and chronic sequences in both *env* and *gag* phylogenetic trees (Figure 1). *Env* sequences from 164 DPI formed a monophyletic cluster within sub-subtype A2, while at 2,048 DPI, one *env* sequence (out of eight) clustered with sub-subtype A1. We examined *env* sequences from three available intervening time points (Figure 4A) and found only the initial sub-subtype A2 strain in a total of 24 sequences (Figure 4B). To confirm the presence of the superinfecting strain, we also examined sequences from a later sample, 2,247 DPI. At this time point, we found *env* sequences from both the sub-subtype A2 strain (*n* = 6) and the sub-subtype A1 strain (*n* = 2) (Figure 4B).

*Gag* sequences from Case 2 at 164 DPI clustered most closely with subtype A2; however, Simplot analysis indicated that the *gag* sequences were a D/A2 recombinant at the initial time point (Figure S2A). At 2,048 DPI, we detected one subtype A1 strain and two D/A1 recombinants (Figures 1 and S2B). To determine whether the recombinant strain was derived from the initial subtype D/A2 strain, we separately analyzed the 5′ subtype D portion of these sequences. Sequences from both time points clustered together on a phylogenetic tree (unpublished data), suggesting that the D/A1 strain present at 2,048 DPI was generated by recombination between the initial subtype D/A2 strain and the novel subtype A1 strain. We did not detect the subtype A1 strain or D/A1 recombinant in *gag* at any of the three intervening time points (25 total sequences) or at the later time point (eight sequences) (Figure 4C). For this individual, we did not have enough HIV-1 DNA available to confirm the timing of superinfection using ssPCR.

As shown in Figure 4D, our results suggest that this individual was initially infected with a subtype D/A2 recombinant (D/A2 in *gag* and A2 in *env*). Between 1,680 and 2,048 DPI, this individual was superinfected with a subtype A1 strain, which was detected in both *env* and *gag*. Within the *gag* region, we also detected a recombinant between the initial and superinfecting strains.

### Case 3 (QB850): Intersubtype Superinfection (Subtype A–D) between 52 and 73 DPI Detected in *env*

For Case 3, *env* sequences from 45 DPI belonged to subtype A, while at 1,768 DPI we detected both subtype A and an A/D recombinant (Figures 1 and S3). In analysis of only the subtype A portion (C2–V3), all sequences formed a monophyletic cluster (unpublished data), suggesting that the new A/D sequence arose by recombination between the initial subtype A strain and a novel subtype D strain.

**Figure 5:**
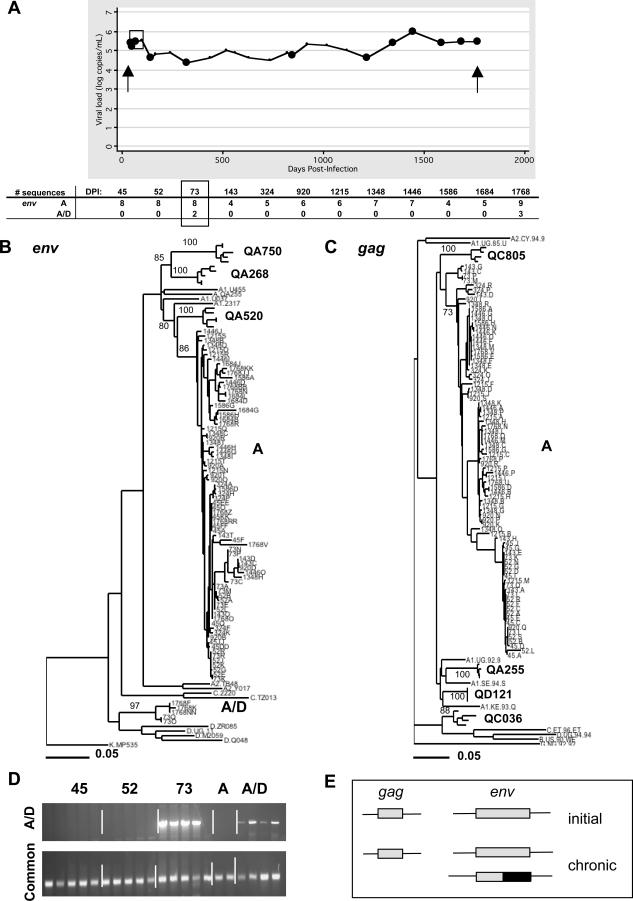
Case 3 (QB850): Intersubtype Superinfection (Subtype A–D) between 52 and 73 DPI Detected in *env* The layout of this figure is similar to that of Figure 3, as described in the legend for Figure 3. (A) Plot of viral load (log RNA copies/mL plasma) versus DPI with the number of sequences of each strain indicated. (B) Maximum-likelihood tree of *env* sequences from all time points. Cluster A contains sequences from all time points, while cluster A/D contains sequences from 73 and 1,768 DPI. (C) Maximum-likelihood tree of *gag* sequences from all time points, illustrating unusually high divergence. (D) Amplification of 50 HIV-1 *env* genome copies from the indicated time points using strain-specific primers (top panel) and common primers (bottom panel). (E) Summary of the strains detected. Light gray = subtype A; black = subtype D.

In analysis of sequences from intervening time points (Figure 5A), we detected only subtype A at 52 DPI, but both A and A/D at 73 DPI (Figure 5B). Forty-four sequences obtained from eight more intervening time points between 73 and 1,768 DPI all belonged to subtype A and clustered with the 45 DPI sequences. Using ssPCR to amplify 50 genome copies, the second strain was not detected at 45 or 52 DPI but was detected at 73 DPI (Figure 5).

*Gag* sequences from 45 and 1,768 DPI belonged to subtype A and formed a monophyletic cluster (Figure 1). The divergence at 1,768 DPI was unusually high: 6.1%, which is more than 3 standard deviations higher than the average divergence for all individuals. This high divergence could be the result of transmission of a highly diverse virus population from the source partner, rapid de novo evolution of the initial strain in this individual, or superinfection by a strain that is closely related to the initial strain. In examination of sequences from intervening time points, we did not detect any sequences that formed a separate cluster on a *gag* phylogenetic tree (Figure 5C).

Together, these results suggest that this individual was initially infected with a subtype A strain and was superinfected between 52 and 73 DPI with a subtype D strain, which recombined with the initial strain within *env* V1–V5 (Figure 5E). After it was first detected at 73 DPI, the A/D recombinant strain was not detected in a total of 44 sequences from eight time points sampled over four and a half years, however it was detected (in three out of 12 sequences) at the last time point examined, 1,768 DPI.

### Case 4 (QD022): Intersubtype Superinfection (Subtype A–C) between 1,832 and 1,957 DPI Detected in *env*

**Figure 6:**
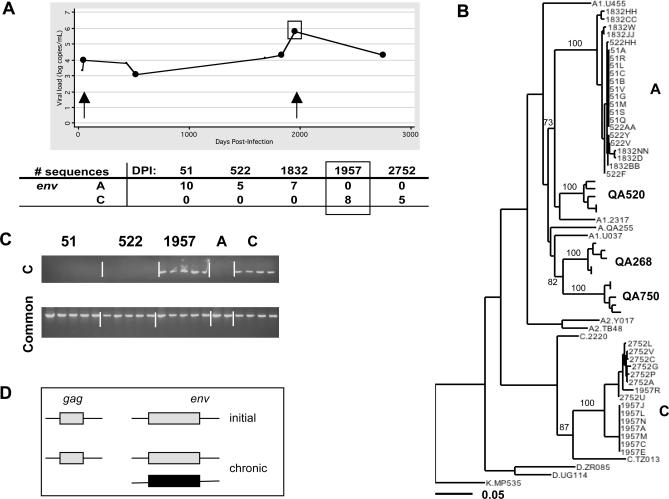
Case 4 (QD022): Intersubtype Superinfection (Subtype A–C) between 1,832 and 1,957 DPI Detected in *env* The layout of this figure is similar to that of Figure 3, as described in the legend for Figure 3. (A) Plot of viral load (log RNA copies/mL plasma) versus DPI with the number of sequences of each strain indicated. B) Maximum-likelihood tree of *env* sequences from all time points. Cluster A contains sequences from 51, 522, and 1,832 DPI, while cluster C contains sequences from 1,957 and 2,752 DPI. (C) Amplification of 50 *env* HIV-1 genome copies from the indicated time points using strain-specific primers (top panel) and common primers (bottom panel). (D) Summary of the strains detected. Light gray = subtype A; black = subtype C.

For Case 4, *env* sequences from 51 DPI belonged to subtype A, while those from 1,957 DPI belonged to subtype C, suggesting intersubtype superinfection (Figure 1). Because this individual had limited follow-up, we were only able to study sequences from two intervening time points (Figure 6A). Sequences from 522 and 1,832 DPI belonged to subtype A and clustered with the 51 DPI sequences (Figure 6B). To confirm presence of the subtype C strain, we also examined sequences from a sample obtained later in infection (2,752 DPI). At this time, all *env* sequences belonged to subtype C and clustered with the 1,957 DPI sequences (Figure 6B). Results from *env* ssPCR supported those from single-copy sequencing; the subtype C strain was not detected at 51, 522, and 1,832 DPI but was detected at 1,957 DPI (Figure 6C and unpublished data).

In the analysis of initial and chronic *gag* sequences, all sequences formed a monophyletic cluster within subtype A (Figure 1). We examined *gag* sequences from intervening time points and did not observe evidence of superinfection (unpublished data). Overall, these results suggest that this individual was initially infected with a subtype A strain and was superinfected with a subtype C strain between 1,832 and 1,957 DPI (Figure 6D). Because we did not detect the novel strain in the *gag* region, it is likely that the two strains recombined, and the strain that predominated contained the initial subtype A in *gag* and the novel subtype C in *env*.

### Case 5 (QA413): Intrasubtype Superinfection (Subtype A) between 714 and 1,007 DPI Detected in *env*

**Figure 7:**
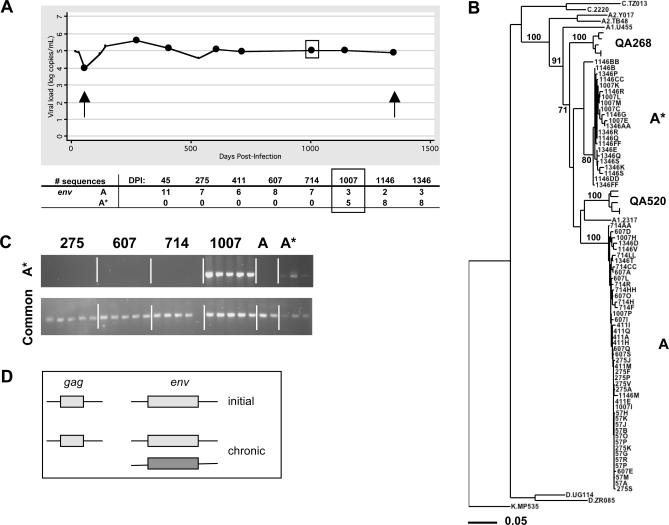
Case 5 (QA413): Intrasubtype Superinfection (Subtype A) between 714 and 1,007 DPI Detected in *env* The layout of this figure is similar to that of Figure 3, as described in the legend for Figure 3. (A) Plot of viral load (log RNA copies/mL plasma) versus DPI with the number of sequences of each strain indicated. (B) Maximum-likelihood tree of *env* sequences from all time points. The cluster of strain A contains sequences from all time points, while the cluster of strain A* contains sequences from 1,007, 1,146, and 1,346 DPI. (C) Amplification of 50 *env* HIV-1 genome copies from the indicated time points using strain-specific primers (top panel) and common primers (bottom panel). (D) Summary of the strains detected. Light gray = subtype A; dark gray = A*.

In this case, *env* sequences from 45 DPI formed a monophyletic cluster, while some sequences from 1,346 DPI formed a separate cluster within subtype A, suggesting intrasubtype superinfection (Figure 1). We examined *env* sequences from six intervening time points (Figure 7A) and first detected the superinfecting strain (A*) at 1,007 DPI (Figure 7B). Using ssPCR, we did not detect strain A* in *env* in 50 genome copies from 57, 275, 411, 607, or 714 DPI but did detect it at 1,007 DPI (Figure 7C and unpublished data).

In examination of *gag* sequences, we did not identify a novel strain in 23 total sequences from 714, 1,007, 1,146, or 1,346 DPI (unpublished data). Based on these results, it appears that this individual was initially infected with a subtype A strain and was superinfected with another subtype A strain between 714 and 1,007 DPI. As in Case 4, our failure to detect the superinfecting strain in the *gag* region suggests that the strains recombined, generating a variant that contained the initial subtype A strain in *gag* and the novel subtype A strain in *env* (Figure 7D).

### Case 6 (QB685): Intrasubtype Superinfection (Subtype A) between 303 and 1,453 DPI Detected in *gag*

**Figure 8:**
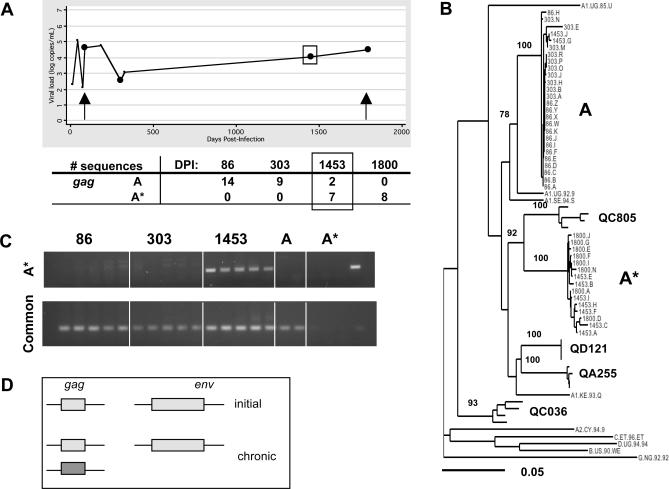
Case 6 (QB685): Intrasubtype Superinfection (Subtype A) between 303 and 1,453 DPI Detected in *gag* The layout of this figure is similar to that of Figure 3, as described in the legend for Figure 3. (A) Plot of viral load (log RNA copies/mL plasma) versus DPI with the number of sequences of each strain indicated. (B) Maximum-likelihood tree of *gag* sequences from all time points. The cluster of strain A contains sequences from all time points except 1,800 DPI. The cluster of strain A* contains sequences from 1,453 and 1,800 DPI. (C) Amplification of 50 *gag* HIV-1 genome copies from the indicated time points using strain-specific primers (top panel) and common primers (bottom panel). (D) Summary of the strains detected. Light gray = subtype A; dark gray = A*.

Intrasubtype superinfection in this case was suggested by separate clustering of initial (86 DPI) and chronic (1,800 DPI) *gag* sequences (Figure 1). Only two intervening samples were available for estimating the timing of superinfection in this case (Figure 8A). The superinfecting strain (A*) was detected at 1,453 but not 303 DPI by single-copy sequencing (Figure 8B). Using ssPCR to amplify 50 genome copies, we also detected A* at 1,453 but not 303 or 86 DPI (Figure 8C).

In the analysis of *env* sequences from Case 6, initial and chronic sequences clustered together on a phylogenetic tree (Figure 1), although the divergence at 1,800 DPI was relatively high (8.2%). Examination of sequences from intervening time points did not reveal the presence of a second strain. These results suggest that this individual acquired a subtype A intrasubtype superinfection between 303 and 1,453 DPI (Figure 8D). Because we did not detect the second strain in the *env* region, it is likely that the two strains recombined (Figure 8D).

### Case 7 (QC885): Intersubtype Superinfection (Subtype C–A) between 58 and 152 DPI Detected in *gag*

*Gag* sequences sampled from Case 7 at 58 DPI belonged to subtype C, while those sampled at 1,405 DPI clustered between subtypes A and C (Figure 1). Simplot analysis of the chronic sequences revealed A/C recombinant sequences (Figure S1). Phylogenetic analysis of the 3′ subtype C portion of these sequences indicated that the novel subtype C was derived from the original subtype C (unpublished data).

**Figure 9:**
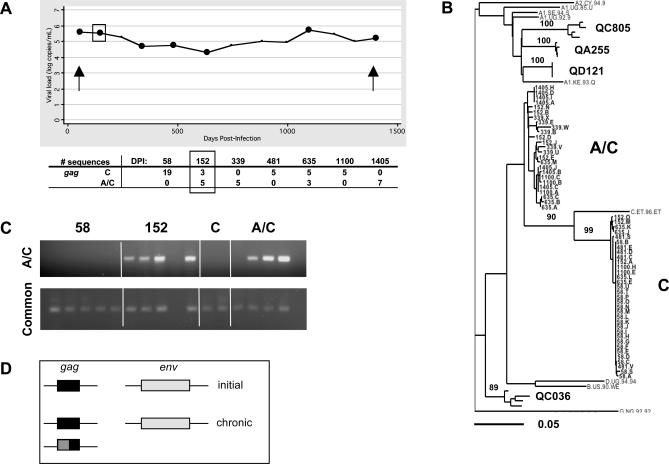
Case 7 (QC885): Intersubtype Superinfection (Subtype C–A) between 58 and 152 DPI Detected in *gag* The layout of this figure is similar to that of Figure 3, as described in the legend for Figure 3. (A) Plot of viral load (log RNA copies/mL plasma) versus DPI with the number of sequences of each strain indicated. (B) Maximum-likelihood tree of *gag* sequences from all time points. The cluster of strain C contains sequences from all time points except 339 and 1,405 DPI. The cluster of strain A/C contains sequences from 152, 339, 635, and 1,405 DPI. (C) Amplification of 50 *gag* HIV-1 genome copies from the indicated time points using strain-specific primers (top panel) and common primers (bottom panel). (D) Summary of the strains detected. Light gray = subtype A; black = subtype C.

To determine when the A/C strain first appeared, we examined sequences from five intervening time points (Figure 9A). The A/C recombinant was detected at the earliest available intervening time point (152 DPI) and several other time points (Figure 9B). Using ssPCR, we did not detect the A/C strain in 50 genome copies from the 58 DPI sample but did detect it at 152 DPI (Figure 9C). In addition, the superinfecting strain was not detected in analysis of *env* sequences from any time point (unpublished data).

As shown in Figure 9D, these results suggest that this individual was initially infected with a C/A recombinant (C in *gag*, A in *env*). This individual acquired a novel subtype A strain between 58 and 152 DPI, which recombined with the initial subtype C strain within the *gag* region studied and was not detected in the *env* region.

### Summary of Superinfection Cases

Overall, we observed seven cases of HIV-1 superinfection among 36 individuals with 188.7 total person-years of follow-up, corresponding to an incidence of 3.7% per person-year. Superinfection occurred throughout the course of infection, with the earliest between 52–73 DPI and the latest between 1,832–1,957 DPI (Table 1). In most cases, the viral load at the time of superinfection was at the expected level for this cohort. The median viral load at the time point before superinfection was 4.5 log copies RNA/mL plasma (range 2.6–5.6), compared to a median viral load set point of 4.5 log copies RNA/mL plasma in this cohort [31]. The median log viral load at the time point after superinfection was 5.1 log copies RNA/mL plasma (range 4.0–5.8). Our sample size was too small for a meaningful statistical analysis of the change in viral load between these time points; however, the change was negligible for five of the seven individuals (median 0.0 log copies RNA/mL plasma, range: −0.2 to 0.2). For the other two individuals (Cases 4 and 6), the viral load increased substantially (by 1.5 log copies RNA/mL plasma) between the time points immediately before and immediately after superinfection (Figures 6A and 8A). CD4 counts were available for five of the seven individuals at the time point after superinfection and in all five cases were greater than 200 cells/uL (Table 1).

Superinfection was detected in analysis of both *env* and *gag* sequences in only two of the seven cases, and in one of these cases the superinfecting strain was detected in both regions only at some time points. In the other five cases, the superinfecting and initial strains recombined. In five cases, both the initial and superinfecting strains persisted throughout infection in at least one genome region, while in the remaining two cases (Cases 4 and 6), the initial strain was not present to our level of detection after superinfection, suggesting that it was substantially replaced by the superinfecting strain.

## Discussion

This study represents the most in-depth population-level assessment of the incidence and timing of HIV-1 superinfection to date. Although the number of individuals included in the study was relatively small, they had extensive long-term follow-up. Importantly, because individuals were enrolled before their first infection and followed for approximately five years after infection, we were able to assess the incidence of superinfection throughout the course of HIV-1 infection. By contrast, many of the previously published cases of superinfection were identified as clinical case reports [3,12,15,16,18,32,33] or in studies with short follow-up time [2,17,21]. In addition, the design of this cohort included frequent longitudinal sampling, and as a result, in most cases we were able to pinpoint the timing of superinfection to within months. These studies indicate that HIV-1 superinfection occurs both during acute infection and during chronic infection, as late as 5 y post-infection. Five out of seven of the superinfections we detected occurred more than a year after infection and while CD4 counts were above 200 cells/uL (before the onset of AIDS and initiation of treatment). In these cases, the immune response to natural infection was not sufficient to protect against re-infection.

We found that superinfection, even during chronic infection, was not limited to viruses that are distantly related. We detected three cases of subtype A intrasubtype superinfection. To our knowledge, these are the first documented cases of intrasubtype superinfection with subtype A. Subtype A is highly prevalent in Africa and is the most common subtype in this cohort, representing ∼80% of initial infections [34]. Therefore, we expected most superinfections to be with subtype A viruses. To have the best chance of detecting intrasubtype superinfections in this study, we selected individuals whose initial infection was with subtype A viruses, based on *env* V1–V3 sequences [34]. However, by examining virus sequences from a second region of the genome (*gag*) during primary infection, we found that nine of the 36 individuals (25%) were initially infected with intersubtype recombinant viruses. This is consistent with prior studies that reported a high prevalence of intersubtype recombinant viruses in Kenya [35–37]. We found that two of the individuals initially infected with intersubtype recombinant viruses acquired superinfections with subtype A viruses. Another two individuals who were initially infected with subtype A viruses became superinfected with subtype C or D viruses. Thus, there were four intersubtype superinfections, which we defined as cases in which the two strains belonged to different subtypes in at least one genome region (*gag* or *env*). Although we detected both intersubtype and intrasubtype superinfection, we did not have sufficient power to compare their rates.

Because we screened two regions of the HIV-1 genome, we were able to identify more cases of superinfection than we would have detected by examining only one region. Specifically, had we only examined *env*, we would have detected five cases, and had we only examined *gag*, we would have detected four cases. We interpret these results to indicate that recombination frequently occurred following superinfection (in five out of seven cases). This is concerning because it suggests that superinfection could provide a mechanism for HIV-1 to rapidly gain fitness. For example, superinfection followed by recombination could allow the rapid acquisition of antiretroviral drug resistance or contribute to immune escape. Moreover, because of the high frequency of recombination that we observed, it is likely that we missed some superinfections that we would have detected by examining a greater proportion of the HIV-1 genome. Specifically, we would have missed cases in which recombination occurred and the superinfecting strain was no longer represented in either *gag* p17 or *env* V1-V5. Similarly, other studies that examined a small proportion of the genome may have underestimated the incidence of HIV-1 superinfection.

It is also possible that we missed some cases of superinfection that occurred transiently, as seen by Yerly et al. [17]. In fact, in one case described here, we detected the superinfecting strain at only two of 12 time points studied. We also could have missed cases in which the superinfecting strain was maintained at a low level. Using a binomial distribution, we calculated that in our initial examination of ∼7 sequences per time point, we had a 79% probability of detecting a strain present at 20% prevalence, a 52% probability of detecting a strain present at 10% prevalence, and only a 30% probability of detecting a strain present at 5% prevalence. Because our initial screen could have missed a strain present at a low level, our estimated incidence of 3.7% per person-year is likely to be an underestimate of the true incidence of HIV-1 superinfection.

It is important to note that, as in all studies of HIV-1 superinfection, it is possible that we have included cases in which the two strains were acquired simultaneously or nearly simultaneously (co-infections) and one strain was maintained in a different compartment or at a low level. However, the results from ssPCR, in which we sampled 50 HIV-1 genomes per time point, argue against this. We calculated that by using ssPCR we had a >99% probability of detecting a strain present at 10% prevalence, a 92% probability of detecting a strain present at 5% prevalence, and a 40% probability of detecting a strain present at 1% prevalence. It is noteworthy that in all cases examined here, as well as three cases of superinfection described previously [1], the results of ssPCR and sequence analyses were absolutely concordant, and both indicated the same timing of superinfection.

The frequency of HIV-1 superinfection that we detected, 3.7% per year, is approximately half the incidence of primary infection in this cohort, ∼8% per year [6]. It is likely that the women in this study, who are sex workers, were exposed to HIV-1 more frequently than women in the general population. However, their level of exposure is lower than in other populations where superinfection and co-infection have been reported [21,38,39]. In the original study from which the group examined here was derived, HIV-negative women reported 1–2 sex partners per week and an 83% frequency of condom use [31]. It is notable that, in the original study cohort, the frequency of unprotected intercourse decreased after HIV-1 infection for women who became HIV-1-infected [40]. Therefore, although the overall incidence of superinfection is lower than that of initial infection, the rate per exposure may be quite similar. Because of the relatively small sample size of this study, we were unable to determine whether either the overall incidence or the rate per exposure was statistically significantly different for superinfection versus initial infection. Large population-based studies, controlling for behavioral and other risk factors, are needed to determine whether there is any role of initial HIV-1 infection in preventing subsequent infections.

Our study clearly demonstrates that HIV-positive individuals are at continued risk of acquiring a second HIV infection. However, it does not provide conclusive information on the clinical consequences of HIV-1 superinfection. Many of the previously published studies have shown an increase in viral load and faster disease progression in superinfected individuals [2,3,12,15–18,21,33,39,41] and dually infected individuals [13]. In two of the HIV-1 superinfection cases described here, viral load increased substantially between the time points before and after superinfection. However, in the other five cases, we did not observe a change in viral load. The fact that we observed no change in viral load in the majority of superinfection cases may be a consequence of our study design; we did not select individuals with viral load change or any other specific characteristics other than adequate follow-up. In addition, we noticed that our ability to consistently detect both viral strains was different for cases with and without viral load change. Specifically, in the two cases in which viral load changed substantially, the superinfecting strains appeared to replace the initial strains (to our limit of detection). In the five cases with no viral load change, both viruses persisted after superinfection. Among the latter cases, it is unclear whether there were some cases in which the superinfecting strain was maintained at a lower level than the initial strain because we could not discern what fraction of the overall virus burden was due to each strain. Quantitative analyses of viral RNA using primers specific for each virus will be needed to better address and define the replication dynamics of each virus over time.

The observed association between viral replacement and change in viral load may suggest that virus fitness plays a role in determining the clinical consequences of superinfection. For example, if an individual is superinfected by a highly fit virus, it may out-compete the initial virus and lead to increased viral load. On the other hand, if both viruses are of approximately equal fitness, both might persist without dramatically changing disease progression. Upon inspection of previously published cases of HIV-1 superinfection, increased viral load was associated with apparent replacement of the initial strain in many cases [1,2,12,14–18,33,41]. Further studies comparing virus fitness in cases of increased viral load versus stable viral load may reveal virus characteristics that contribute to the occurrence and outcome of HIV-1 superinfection.

Including the cases presented here, there are now more than 30 documented cases of HIV-1 superinfection, which demonstrate that infection with HIV-1 does not invariably confer protection against later exposures to the virus. Our study illustrates that this lack of protection appears to be largely independent of the timing of re-exposure and the relatedness of the virus strains. If the lack of protection against re-infection is common to all HIV-infected individuals, it calls into question the likelihood of success of an HIV-1 vaccine that is predicated on mimicking the immune response to natural infection. However, HIV-1 superinfection cases differ from a vaccine-challenge setting because individuals' immune responses may be impaired by the first HIV-1 infection. Furthermore, the fact that some HIV-infected individuals become superinfected while others do not could be the result of specific deficits in the immune response among individuals who become superinfected. For example, there is some evidence that individuals who develop a weak neutralizing antibody response to their first infection may be more susceptible to superinfection [26]. By contrast, in several previously published cases, the presence of a CTL response did not protect individuals from superinfection or abrogate the growth of the superinfecting strain [12,16,18,21]. Clarifying the roles of these and other immune factors in protection from HIV-1 superinfection may provide insight into correlates of immune protection against HIV-1 infection in general and may reveal improved markers for assessing vaccine candidates.

Together with a previous study in this cohort [1], we have identified ten cases of HIV-1 superinfection among 56 individuals. These studies demonstrate that both intersubtype and intrasubtype superinfection occur frequently and throughout the course of HIV-1 infection. The cases identified provide an excellent opportunity to compare the immune responses in individuals who become superinfected to those who do not. These studies may help to shape our understanding of immune protection from sexual transmission of HIV-1. In addition, they will provide insight into whether the lack of immune protection indicated by HIV-1 superinfection is a universal phenomenon or a consequence of specific host immune deficits. Distinguishing between these possibilities will help to clarify the implications of superinfection for the success of HIV-1 vaccines.

## Materials and Methods

### Study population.

The individuals in this study were part of a previously described prospective cohort of high-risk HIV-1 seronegative women from Mombasa, Kenya [42]. Blood samples were obtained approximately every month and tested for infection by HIV-1 serology. Over the period when samples were collected for this study, 1993–2004, the average seroincidence in the cohort was 8% per year [6]. For individuals who seroconverted, the estimated date of infection was determined by testing stored plasma samples from prior to seroconversion for HIV-1 RNA [31]. HIV-1-infected individuals continued to be followed with blood samples collected approximately every 3 mo. Beginning in 1998, absolute CD4 counts were determined for all infected individuals [6]. All infected individuals acquired HIV-1 through heterosexual contact [31]. The individuals in this cohort had one to two sex partners per week; they engaged in sex work to supplement income, but typically not as a primary source of support [43]. None of the women reported using antiretroviral therapy during follow-up for the present study, although some have since started therapy due to CD4 counts <200/uL. Thirty-six individuals were included in this study based on the following criteria: infection with a subtype A virus based on *envelope* V1–V3 sequences [34], availability of a blood sample from within the first year of infection, and available samples for approximately 5 y post-infection. Informed consent was obtained from all participants. The study was approved by the ethical review committees of the University of Nairobi, the University of Washington, and the Fred Hutchinson Cancer Research Center.

### Molecular analyses.

HIV-1 proviral DNA sequences were obtained from two time points for each of the 36 individuals: the first visit after the detection of seroconversion and a visit during chronic infection (approximately 5 y later). For individuals identified as potential superinfection cases, samples were also analyzed from intervening follow-up visits. DNA was extracted from ∼5 million viable frozen PBMCs using the QIAamp DNA Blood Mini kit (Qiagen).

For each sample, the HIV-1 proviral copy number was estimated using real-time quantitative PCR to amplify a 165-bp region of *pol* [44,45]. From an estimated one copy of proviral template, separate nested PCRs were used to amplify two regions of the HIV-1 genome: *envelope* V1–V5 (∼1.2 kb) and *gag* p17 (∼700 bp). PCR primers and conditions are described in Table S1 (*env*) and Table S2 (*gag*). For each sample, at least 24 independent PCRs were performed. The PCR products were treated with Exo-SAP (Amersham Biosciences) and directly sequenced.

### Phylogenetic analysis.

Sequences were assembled using Sequencher (Gene Codes) and automatically aligned using Clustal X [46]. Alignments were manually edited using MacClade 4.01 [47], and regions that could not be unambiguously aligned (mainly regions of *env* V1/V2 and V4) were removed from analysis. We also removed virus sequences that were identified as hypermutated, using a modified version of the method used by Pace et al. [48].

Phylogenetic trees were constructed by two methods: neighbor-joining using an HKY85 model in PAUP* [49] and maximum-likelihood (ML) using a general time reversible model in GARLI 0.95 [50]. Both methods yielded comparable results, and only the ML trees are shown. Trees were prepared for publication, including coloring branches and removing branch names using TreeDyn [51]. For each individual, the MRCA of the sequences from the earliest available sample was reconstructed using maximum likelihood in GARLI. Genetic distances were calculated using a general time reversible model in PAUP*. Genetic diversity was defined as the average pairwise distance between sequences. Divergence was defined as the maximum pairwise distance between a sequence and the MRCA of the same individual. For each pair of individuals, the between-individual divergence was calculated as the maximum pairwise distance between a sequence from one individual and the MRCA of the other individual.

Virus subtypes were determined using the NCBI genotyping tool [52] and by clustering on phylogenetic trees with reference sequences from the Los Alamos National Laboratory HIV database (http://www.hiv.lanl.gov/). Intersubtype recombinant sequences were characterized using Simplot [53]; sequences were compared to reference sequences of known subtype obtained from the LANL database.

### ssPCR.

 For each case of superinfection, primers were designed to specifically amplify the second (superinfecting) strain. For each region (*gag* and *env*), common primers were also designed to amplify all strains in the superinfection cases. Primers and PCR conditions are listed in Tables S1 (*env*) and S2 (*gag*). PCR products representing the first and second strains in each superinfection case were cloned into a TOPO 2.1 vector and amplified with the common and specific primers. In each case, the specific primers could amplify one copy of the superinfecting strain, but not 10^3^ copies of the initial strain. Common primers could amplify all strains using one copy of template. As expected, amplifications from an estimated single copy of template were successful approximately 1/3–1/2 of the time. DNA from PBMCs was amplified using a nested PCR with common primers in the first round and either common or strain-specific primers in the second round.

## Supporting Information

Figure S1 Simplot Analysis of *gag* Sequences from Case 7 A sequence from 1,405 DPI was compared to reference sequences of known subtype from the LANL database.
(43 KB PDF)

Figure S2 Simplot Analysis of *gag* Sequences from Case 2 Sequences from 164 DPI (A) and 2,048 DPI (B) were compared to reference sequences of known subtype from the LANL database.
(81 KB PDF)

Figure S3 Simplot Analysis of *env* Sequences from Case 3 A sequence from 1,768 DPI was compared to reference sequences of known subtype from the LANL database.
(45 KB PDF)

Table S1 *Envelope* PCR Primers Cycling conditions for all PCRs were: 94 ° × 5 min; 35 × (94 ° × 1 min, annealing temp × 1 min, 72 ° × 3 min); 72 ° × 8 min.
(43 KB DOC)

Table S2 *Gag* PCR Primers Cycling conditions for all PCRs were: 94 ° × 5 min; 25 × (94 ° × 1 min, annealing temp × 30 s, 72 ° × 1 min); 72 ° × 6 min.
(41 KB DOC)

### Accession Numbers

Sequences from this study available on GenBank (http://www.ncbi.nlm.nih.gov/Genbank/) are *gag* sequences (EU158368-EU158771) and *env* sequences (EU163983-EU164399).

## Abbreviations

CTL: cytotoxic T lymphocyte
DPI: days post-infection
MRCA: most recent common ancestor
ssPCR: strain-specific polymerase chain reaction
