# Supplementary material for: Chronic HIV-1 Infection Frequently Fails to Protect against Superinfection
Source: PLoS Pathog. 2007 Nov 16;3(11):e177. doi: 10.1371/journal.ppat.0030177 (PMC2077901; doi:10.1371/journal.ppat.0030177)
Supplement: Supplementary file 1 [file ppat.0030177.sg001.pdf]

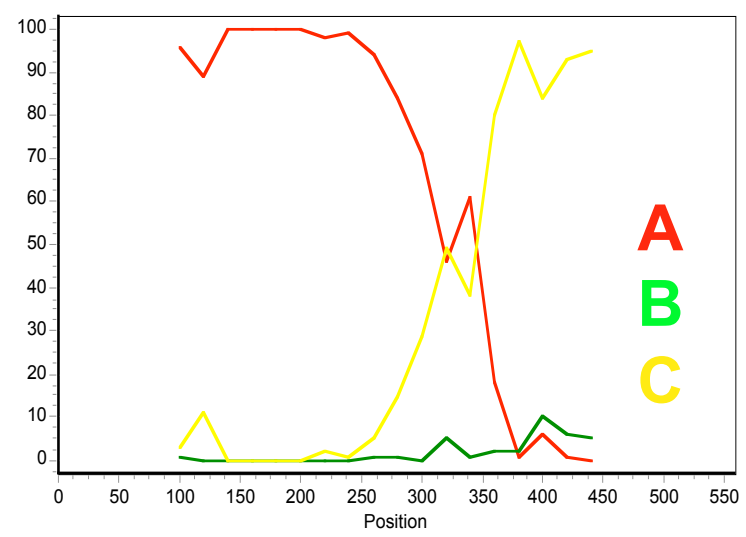

100
90
80
70
60
50
40
30
20
10
0
0
50
100
150
200
250
300
350
400
450
500
550
Position
A
B
C
