# Supplementary material for: Chronic HIV-1 Infection Frequently Fails to Protect against Superinfection
Source: PLoS Pathog. 2007 Nov 16;3(11):e177. doi: 10.1371/journal.ppat.0030177 (PMC2077901; doi:10.1371/journal.ppat.0030177)
Supplement: Supplementary file 2 [file ppat.0030177.sg002.pdf]

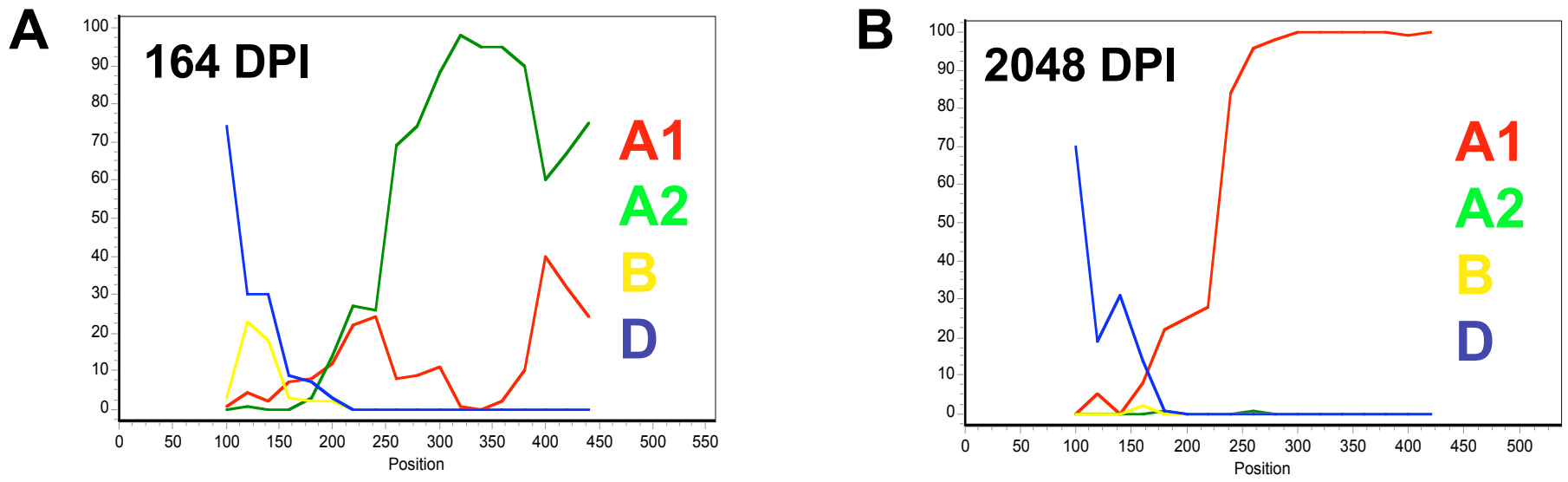

A
164 DPI
A1
A2
B
D
Position
B
2048 DPI
A1
A2
B
D
Position
