# Supplementary material for: Chronic HIV-1 Infection Frequently Fails to Protect against Superinfection
Source: PLoS Pathog. 2007 Nov 16;3(11):e177. doi: 10.1371/journal.ppat.0030177 (PMC2077901; doi:10.1371/journal.ppat.0030177)
Supplement: Supplementary file 3 [file ppat.0030177.sg003.pdf]

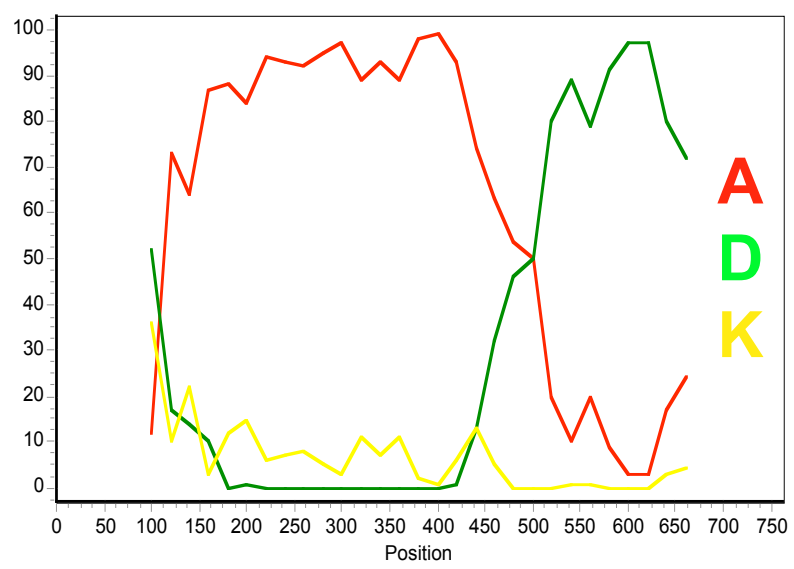
100
90
80
70
60
50
40
30
20
10
0
0 50 100 150 200 250 300 350 400 450 500 550 600 650 700 750
Position
A
D
K
