# Supplementary material for: Chronic HIV-1 Infection Frequently Fails to Protect against Superinfection
Source: PLoS Pathog. 2007 Nov 16;3(11):e177. doi: 10.1371/journal.ppat.0030177 (PMC2077901; doi:10.1371/journal.ppat.0030177)
Supplement: Supplementary file 4 [file ppat.0030177.st001.doc]

| PCR | Product Size | Primers | Annealing Temp |
| --- | --- | --- | --- |
| *env* V1-V5  Round 1 | 1415 bp | 5' TTGCAATAGAAAAATTCTCCTC 3'  (HXB2: 6310-6332)  5' CCTGGTGGGTGCTACTCCTA 3'  (HXB2: 7724-7705) | 50^0^ |
| *env* V1-V5  Round 2  AND  *env* ssPCR  Round 1 | 1098 bp | 5' CCATGTGTAAAGTTAACCCC 3'  (HXB2: 6576-6595)  5' ATGAGGGACAATTGAGAAGTGTCTAG 3'  (HXB2: 7647-7673) | 55^0^ |
| *env* ssPCR  Round 2  Common | 570 bp | 5' GTAATACCTCAGCCATTAC 3'  (HXB2: 6811-6829)  5' TTGCAATAGAAAAATTCTCCTC 3'  (HXB2: 7360-7381) | 55^0^ |
| *env* ssPCR  Round 2 Case 1 | 771 bp | 5' CCACTATCACCACCAATGTCACCACTATC 3'  (HXB2: 6772-6801)  5' CATTTGACTCTAGGCTGCTTGAATTTGTG 3'  (HXB2: 7514-7543) | 55^0^ |
| *env* ssPCR  Round 2 Case 3 | 364 bp | 5' TACACAGTTGATTAGTCCTGC 3'  (HXB2: 6965-6985)  5' CATCCTCGGGAGGGGACC 3'  (HXB2: 7312-7329) | 60^0^ |
| *env* ssPCR  Round 2  Case 4 | 781 bp | 5' CCAACAGCACTAGTGATAATAG 3'  (HXB2: 7508-7529)  5' CTCTCATTAAACAATTCACTATG 3'  (HXB2: 8265-8289) | 55^0^ |
| *env* ssPCR  Round 2 Case 5 | 795 bp | 5' CAGCAACCCGAACATGACAG 3'  (HXB2: 7191-7210)  5' CTGGCAGTCTCATTATTCCTAG 3'  (HXB2: 7965-7986) | 60^0^ |
