# Supplementary material for: Chronic HIV-1 Infection Frequently Fails to Protect against Superinfection
Source: PLoS Pathog. 2007 Nov 16;3(11):e177. doi: 10.1371/journal.ppat.0030177 (PMC2077901; doi:10.1371/journal.ppat.0030177)
Supplement: Supplementary file 5 [file ppat.0030177.st002.doc]

| PCR | Product Size | Primers | Annealing Temp |
| --- | --- | --- | --- |
| *gag* p17  Round 1 | 1011 bp | 5' CACTGCTTAAGCCTCAATAAAGCTTGCCTT 3'  (HXB2: 512-541)  5' TCCTGAACGGTACTAGTAGTTCCTGCTA 3'  (HXB2:1496-1523) | 55^0^ |
| *gag* p17  Round 2 | 704 bp | 5' GCGAGAGCGTCAGTATTAAG 3'  (HXB2:796-815)  5' TGCTATGTCACTTCCCCTTGGTTCTCT 3'  (HXB2:1474-1500) | 55^0^ |
| *gag* ssPCR  Round 1 | 650 bp | 5' GGGAAAAAATTCGGTTAAGGCCAGGGG 3'  (HXB2:836-862)  5' CCCTTGGTTCTCTCATCTGGCCTG 3'  (HXB2:1423-1486) | 66^0^ |
| *gag* ssPCR  Round 2 Common | 150 bp | 5' AATGCATGGGTAAAAGTAATAGA 3'  (HXB2:1246-1269)  5' CTTTTAACATTTGCATAGCTGC 3'  (HXB2:1375-1396) | 60^0^ |
| *gag* ssPCR  Round 2 Case 1 | 216 bp | 5' AACTACCCTATAGTGCAAAATGC 3'  (HXB2:1180-1203)  5' CTTTTAACATTTGCATAGCTG 3'  (HXB2:1375-1396) | 60^0^ |
| *gag* ssPCR  Round 2  Case 6 | 213 bp | 5' TACCCTATAGTGCGAAATGTG 3'  (HXB2:1183-1204)  5' CTTTTAACATTTGCATAGCTGC 3'  (HXB2:1375-1396) | 60^0^ |
| *gag* ssPCR  Round 2  Case 7 | 147 bp | 5' AGCCGCTGATAGTAGCC 3'  (HXB2:1249-1265)  5' CTTTTAACATTTGCATAGCTGC 3'  (HXB2:1375-1396) | 60^0^ |
